# Supplementary material for: Heat adaptation of phage T7 under an extended genetic code
Source: Virus Evol. 2021 Dec 1;7(2):veab100. doi: 10.1093/ve/veab100 (PMC8923235; doi:10.1093/ve/veab100)
Supplement: veab100_Supp [file veab100_supp.zip › Sequencing_Analysis.zip!/Sequencing_Analysis/A1/evidence/SNP_12.html]

BRESEQ :: Evidence


| Predicted mutation | | | | | | |
| --- | --- | --- | --- | --- | --- | --- |
| evidence | seq id | position | mutation | annotation | gene | description |
| RA | NC\_001604.1 | 35,095 | A→G | intergenic (–/–) | – / – | –/– |

| Read alignment evidence... | | | | | | | | | | | |
| --- | --- | --- | --- | --- | --- | --- | --- | --- | --- | --- | --- |
|  | seq id | position | | ref | new | freq | score (cons/poly) | reads | annotation | genes | product |
| \* | NC\_001604.1 | 35,095 | 0 | A | G | 98.9% | 7805.7 / NA | 2579 | intergenic (–/–) | *–/–* | –/– |
| Reads supporting (aligned to +/- strand):  **ref** base A (7/11);  **major** base G (1212/1314);  **minor** base T (15/13);  **total** (1240/1339) | | | | | | | | | | | |
| Fisher's exact test for biased strand distribution *p*-value = 5.74e-01 | | | | | | | | | | | |
| Kolmogorov-Smirnov test that lower quality scores support variant *p*-value = 6.63e-01 | | | | | | | | | | | |

Only 100 of 2579 total aligned reads displayed.  

`CTCTCTGACAAAGGCTTGGGGTCCAGCCGATGGCTACACGACCATCGAGTTACGTCGAGTAACCTCCACTACCGACCGATTGGTTGACTTTACGGATGGTTCAATCCTCCGCGCGTATGACCTTAACGTCGCTCAGATTCAAACGATGCACGTAGCGGAAGAGGCCCGTGACCTCACTACGGATACTATCGGTGTCAATAACGATGGTCACTTGGATGCTCGTGGTCGTCGAATTGTGAACCTAGCGAACGCCGTGGATGACCGCGATGCTGTTCCGTTTGGTCAACTAAAGACCATGAACCAGAACTCATGGCAAGCACGTAATGAAGCCTTACAGTTCCGTAATGAGGCTGAGACTTTCAGAAACCAAGCGGAGGGCTTTAAGAACGAGTCCAGTACCAACGCTACGAACACAAAGCAGTGGCGCGATGAGACCAAGGGTTTCCGAGACGAAGCCAAGCGGTTCAAGAATACGGCTGGTCAATACGCTACATCTGCTGGGAACTCTGCTTCCGCTGCGCATCAATCTGAGGTAAACGCTGAGAACTCTGCCACAGCATCCGCTAACTCTGCTCATTTGGCAGAACAGCAAGCAGAC  >  NC_001604.1/34797‑35394`  

                                                                                                                                                                                                                                                                                                          `|`                                                                                                                                                                                                                                                                                                             
  `ctctGACAAAGGCTTGGGGTCCAGCCGATGGCTACACGACCATCGAGTTACGTCGAGTAACCTCCACTACCGACCGATTGGTTGACTTTACGGATAGTTCAATCCTCCGCGCGTATGACCTTAACGTCGCTCAGATTCAAACGATGCACGTAGCGGAAGAGGCCCGTGACCTCACTACGGATATTATCGGTGTCAATAACGATGGTCACTTGGATGCTCGTGGTCGTCGAATTGTGCACCTAGCGAACGCCGTGGATGACCGCGATGCTGTTCCGTTTGGTCAACTAAAGACCATGGACCa                                                                                                                                                                                                                                                                                                         >  1:86267/1‑301 (MQ=255)`  
   `tctGACAAAGGATTGGGGGCCAGACGATGGCGACACGACCATCGAGTTACGGCGAGTAACCTCCACTACCGACCGATTGGTTTACTTTACGGATGGTTAAATCCTCCGCGCGTATGACATTAACGTCGCTCAGATTCAAACGATGCACGTAGCGGAAGAGGCCCGTGAACTCACTACGGATATTATCGGTGTCAATAACGATGGTCACTTGGATGCTCGTGGTCGTCGAATTGTGAACCTAGCTAACGCCGTGGATGACCGCGATGCTGTTCCGTTTGGTCAACTAAAGACCATGGACCAg                                                                                                                                                                                                                                                                                                        <  1:281539/301‑1 (MQ=255)`  
      `gACAAAGGCTTGGGGTCCAGCCGATGGCTACACGACCATCGAGTTACGTCGAGTAACCTCCACTACCGACCGATTGGTTGACTTTACGGATGGTTCAATCATCCGCGCGTATGACCTTAACGTCGCTCAGATTCAAACGATGCACGTAGCGGAAGAGGCCCGTGACCTCACTACGGATATTATCGGTGTCAATAACGATGGTCACTTGGATGCTCGTGGTCGTCGAATTGTGAACCTAGCGAACGCCGTGGATGACCGCGATGCTGTTCCGTTTGGTCAACTAAAGACCATGGACCAGAAc                                                                                                                                                                                                                                                                                                     >  1:5942/1‑301 (MQ=255)`  
        `acagggCTGGTGGTCCAGCCGAGGGCTACACGACCATCGAGTTACGTCGAGTAACCTCCACTACCGACCGATTGGTTGACTTTACGGATGGTTCAATCCTCCGCGCGTATGACCTTAACGTCGCTCAGATTCAAACGATGCACGTAGCGGAAGAGGCCCGTGACCTCACTACGGATATTATCGGTGTCAATAACGATGGTCACTTGGATGCTCGTGGTCGTCGAATTGTGAACCTAGCGAACGCCGTGGATGACCGCGATGCTGTTCCGTTTGGTCAACTAAAGACCATGGACCAGAACTc                                                                                                                                                                                                                                                                                                   <  1:347787/297‑1 (MQ=255)`  
            `ggCTTGGGGTCCGGCCGATGGCTACACGACCATCGAGTTACGTCGAGTAACCTCCACTACCGACCGATTGGTTAACTTTACGGATGGTTCAATCCTCCGCGCGTATGACCTTAACGTCGCTCAGATTCAAACGATGCACGTAGCGGAAGAGGCCCGTGACCTCACTACGGATATTATCGGTGTCAATAACGATGGTCACTTGGATGCTCGTGGTCGTCGAATTGTGAACCTAGCGAAAGCCGTGGATGACCGCGATGCTGTTCCGTTTGGTCAACTAAAGACCATGGACCAGAAcctgtcc                                                                                                                                                                                                                                                                                               >  1:312832/1‑295 (MQ=255)`  
            `ggCTTGGGGTCCAGCCGATGGCTACACGACCATCGAGTTACGTCGAGTAACCTCCACTACCGACCGATTGGTTGACTTTACGGATGGTTCAATCCTCCGCGCGTATTACCTTAACGTCGCTCAGATTCAAACGATGCACGTAGCGGAATAGGCCCGTGACCTCACTACGGATATTATCGGTGTCAATAACGATGGTCACTTGGATGCTCGTGGTCGTCGAATTGTGAACCTAGCGAACTCCGTGGATGACCTCGATGCTGTTCCGTTTGGTCAACTAAAGACCATGGACCAGTACTCCTgt                                                                                                                                                                                                                                                                                               >  1:206534/1‑300 (MQ=255)`  
                `acgagacaggCCGATGGCTACACGACCATCGAGTTACGTCGAGGAACCTCCACTACCGACCGACTGGTTGACTTTACGGATGGTTCAATGCTACGCGGGTATGACCTTAACGTCGCTCAGATTCAAACAATGCACGTAGCGGAAGAGGCCCGTGACCTCACTACGGATATTATCGGTGTCAATAACGATGGTCACTTGGATGCTCGTGGTCGTCGAATTGTGAACCTAGCGAACGCCGTGGATGACCGCGATGCTGTTCCGTTTGGTCAACTAAAGACCATGGACCAGAACTCATGGCAAg                                                                                                                                                                                                                                                                                           <  1:291102/292‑1 (MQ=255)`  
                         `gCCGATGGCTACACGACCATCGAGTTACGTCGAGTAACCTCCACTACCGACCGATTGGTTGACTTTACGGATGGTTCAATCCTCCTCGCGTATGACCTTAACGTCGCTCAGATTCAAACGATGCACGTAGCGGAAGAGGCCCGTGACCTCACTACGGATATTATCGGTGTCAATAACAATGGTCACTTGGATGCTCGTGGTCGTCGAATTGTGAACCTAGCGAACTCCGTGGATGACCGCGATGCTGTTCCGTTTGGTCAACTAAAGACCATGGACCAGAACTCCTGGCAAGCACGTACTg                                                                                                                                                                                                                                                                                  >  1:327413/1‑301 (MQ=255)`  
                         `gCCGATGCCTACACGACCATCGAGTTACGTCGAGTAACCTCCACTACCGACCGATTGGTTGACTTTACGGATGGTTCAATCCTCCGCGCGTATGACCTTAACGTCGCTCAGATTCAAACGATGCACGTAGCGGAAGAGGCCCGTGACCTCACTACGGATATTATCGGTGTCAATAACGATGGTCACTTGGATGCTCGTGTTCGTCGAATTGTGAACCTAGCGAACGCCGTGGATGACCGCGATGCTGTTCCGTTTGGTCAACTAAAGACCATGGACCAGAACTCATGGCAAGCACGTAATg                                                                                                                                                                                                                                                                                  <  1:121952/301‑1 (MQ=255)`  
                          `gagacagCTACACGACCATCGAGTTACGTCGAGTAACCTCCACTACCGACCGATTGGTTGACTTTACGGATGGTTCAATCCTCCGCGCGTATGACCTTAACGTCGCTCAGATTCAAACGATGCACGTAGCGGAAGAGGCCCGTGACCTCACTACGGATATTATCGGTGTCAATAACGATGGTCACTTGGATGCTCGTGGTCGTCGAATTGTGAACCTAGCGAACGCCGTGGATGACCGCGATGCTGTTCCGTTTGGTCAACTAAAGACCATGGACCAGAACTCATGGCAAGCACGTAATGa                                                                                                                                                                                                                                                                                 <  1:156402/295‑1 (MQ=255)`  
                          `ccgtgggCTACACAACCATCGAGTTACGTCAAGTAACCTCCACTACCGACCGATTGGTTAACTTTACGGATGGTTCAATCCTCCGGGCGTATGACCTTAACGTCGCTCAGATTCAAACGAGGCACGTAGAGGAAGGGGATCGTGACCTCACTATGGATAGTATCGGTTTCAATAACGATGGTCACTTGGATGCTCGGGGTCGTCGAATTGGGAACCTAGCGAACGCCGTGGATGACCGCGATGCTGTTCCGTTTGGTCAACTAAAGACCATGGACCAGAACTCATGGCAAGCACGTAATGa                                                                                                                                                                                                                                                                                 <  1:303891/296‑1 (MQ=255)`  
                                 `cTACACGACCATCGAGTTACGTCGAGTAACCTCCACTACCGACCGATTGGTTAACTTTACGGATGGTTCAATCCTCCGCGCGTATGACCTTAACGTCGCTCAGATTCAAACGATGCACGTAGCGGAAGAGGCCCGGGACCTCACTACGGATACTATCGGTGTGAATAACGATGGTCACTTGGATGCTCGTGGTCGCTGAATTGTGAGCCTAGCGAACGCCGTGGTTGACCGCGTTACTGTTCCGTTTGGTCAACTAAAGACCATGGACCAGAACTCATGGCATACCCGTAAtctttccctt                                                                                                                                                                                                                                                                          >  1:286945/1‑292 (MQ=255)`  
                                    `cacGACCATCGAGTTACGTCGAGTAACCTCCACTACCGACCGATTGGTTGACTTTACGGATGGTTCAATCCTCCGCGCGTATGACCTTAACGTCGCTCAGATTCAAACGATGCACGTAGCGGAAGAGGCCCGTGACCTCACTACGGATATTATCGGTGTCAATAACGATGGTCACTTGGATGCTCGTGGTCGTCGAATTGTTAACCTAGCGAACGCCGTGGATGACCGCGATGCTGTTCCGTTTGGTCAACTAAAGACCATGGTCCAGAACTCATGGCAAGCACGTAATGAAGCCTTACAg                                                                                                                                                                                                                                                                       <  1:106808/301‑1 (MQ=255)`  
                                    `cacGACCATCGAGTTACGTCGAGTAACCTCCACTACCGACCGATTGGTTGACTTTACGGATGGTTCAATCCTCCGCGCGTATGACCTTAACGTCGCTCAGATTCAAACGATGCACGTAGCGGAAGAGGCCCGTGACCTCACTACGGATATTATCGGTGTCAATAACGATGGTCACTTGGATGCTCGTGGTCGTCGAATTGTGAACCTAGCGAACGCCGTGGATGACCGCGATGCTGTTCCGTTTGGTCAACTAAAGACCATGGACCAGAACTCATGGCAAGCACGTAATGAAGCCTTACAg                                                                                                                                                                                                                                                                       <  1:115646/301‑1 (MQ=255)`  
                                    `cacGACCATCGAGTTACGTCGAGTAACCTCCACTACCGACCGATTGGTTAACTTTACGGATGGTTCAATCCTCCGAGCGTATGACCTTAACGTCGCTCAGATTCAAACGATGCACGTAGCGGAAGAGGCCCGTGACCTCACTACGGATATTATCGGTGTCAATAACGATGGTCACTTGGATGCTCGTGGTCGTCGAATTGTGAACCTAGCGAACGCCGTGGATGACCGCGATGCTGTTCCGTTTGGTCAACTAAAGACCATGGACCAGAACTCATGGCAAGCACGTAATGAAGCCTTACAg                                                                                                                                                                                                                                                                       <  1:72415/301‑1 (MQ=255)`  
                                        `aCCATCGAGTTACGTCGAGTAACCTCCACTACCGACCGATTGGTTGACTTTACGGATGGTTCAATCCTCCGCGCGTATGACCTTAACGTCGCTCAGATTCAAACGATGCACGTAGCGGAAGAGGCCCGTGACCTCACTACGGATATTATCGGTTTCAATAACGATGGTCACTTGGATGCTCGTGGTCGTCGAATTGTGAACCTAGCTAACGCCGTTGCTGACCGCGATGCTGTTCCGTTTGGTCAACTAAAGACCATGGACCAGAACTCATGGCAAGCCCGTAATGAAGccctgcttcttt                                                                                                                                                                                                                                                                   >  1:241219/1‑291 (MQ=255)`  
                                        `aCCATCGAGTTACGTCGAGTAACCTCCACTACCGACCGATTGGTTGACTTTACGGATGGTTCAATCCTCCGCGCGTATGACCTTAACGTCGCTCAGATTCAAACGATGCACGTAGCGGAAGAGGCCCGTGACCTCACTACGGATATTATCGGTGTCAATAACGATGGTCACTTGGATGCTCGTGGTCGTCGAATTTTGAACCTAGCGAACGCCGTGGATGACCGCGATGCTGTTCCGTTTGGTCAACTAAAGACCATGGACCAGAACTCATGGCAAGCCCGTAATGAAGCCTTACAGTTcc                                                                                                                                                                                                                                                                   >  1:11903/1‑301 (MQ=255)`  
                                          `cagcgtcagatgtgtataagagacagcTACCGACCGATTGGTTGACTTTACGGATGGTTCAATCCTCCGCGCGTATGACCTTAACGTCGCTCAGATTCAAACGATGCACGTAGCTGAAGAGGCCCGTGACCTCACTACGGATATTATCGGTGTCAATAACGATGGTCACTTGGATGCTCGTGGTCGTCGAATTGTGAACCTAGCGAACGCCGTGGATGACCGCGATGCTGTTCCGTTTGGTCAACTAAAGACCATGGACCAGAACTCATGGCAAGCACGTAATGAAGCCTTACAGTTCCGt                                                                                                                                                                                                                                                                 >  1:82603/27‑301 (MQ=255)`  
                                           `aTCGAGTTACGTCGAGTAACCTCCACTACCGACCGATTGGTTGACTTTACGGATGGTTCAATCCTCCGCGCGTATGACCTTAACGTCGCTCAGATTCAAACGATGCACGTAGCGGAAGAGGCCCGTGACCTCACTACGGATATTATTGGTGTCAATAACGATGGTCACTTGGATGCTCGTGGTCGTCGAATTGTGAACCTAGCGAACGCCGTGGATGACCGCGATGCTGTTCCGTTTGGTCAACTAAAGACCATGGACCAGAACTCATGGCAAGCACGTAATGAAGCCTTACAGTTCCGTa                                                                                                                                                                                                                                                                >  1:248994/1‑301 (MQ=255)`  
                                            `tCGGGTTACGTCGAGTAACCTCCACTAGCGACCGATTGGTTGACTTTACGGATGGTTCAATCCTCCGCGCGTATGACCGTAACGTCGCTCAGATTCAAACGATGCACGTAGCGGAAGAGGCCCGTGACCTCACTACGGATATTATCGGTGTCAATAACGATGGTCACTTGGATGCTCGTGGTCGTCGAATTGTGAACCTAGCGAACGCCGTGGATGACCGCGATGCTGTTCCGTTTTGTCAACTAAAGACCATGGACCAGAACTCATGGCAAGCACGTAATGAAGCCTTACAGTTCCGTaa                                                                                                                                                                                                                                                               <  1:146608/301‑1 (MQ=255)`  
                                            `aggAGTTACGTCGAGTAACCTCCACGACCGCCCGATTGGTTGACGTTACGGATGGGTCAATCCTCCGCGCGTATGACCTTAACGTCGCTCAGATTCAAACGATGCACGTAGCGGAAGAGGCCCGTGACCTCACTACGGATATTATCGGTATCAATAACGATGGTCACTTGGATGCTCGTGGTCGTCGAATTGTGAACCTAGCGAACGCCGTGGATGACCGCGATGCTGTTCCGTTTGGTCAACTAAAGACCATGGACCAGAACTCATGGCAAGCACGTAATGAAGCCTTACAGTTCCGTaa                                                                                                                                                                                                                                                               <  1:197504/299‑1 (MQ=255)`  
                                                   `aCGTCGAGTAACCTCCACTACCGACCGATTGGTTGACTTTACGGATGGTTCAATCCTCCGCGCGTATGACCTTAACGTCGCTCAGATTCAAACGATGCACGTAGCGGAAGCGGCCAGTGACCTCACTACGGATATTATCGGTGTCAATAACGATGGTCACTTGGATGCTCGTGTTCGTCGAATTGTGAACCTAGCGAACGCCTTGGATGACCGCGATGCTGTTCCGTTTGGTCAACTAAAGACCATGGACCAGAACTCATGGCAAGCACGTAATGAAGCCTTACAGTTCCGTaactgtctc                                                                                                                                                                                                                                                        >  1:46747/1‑294 (MQ=255)`  
                                                        `gAGTAACCTCCACTACCGACCGATTGGTTGACTTTACGGATGGTTCAATCCTACGCGCGTATGACCTTAACGTCGCTCAGATTCAAACGATGCACGTAGCGGAAGAGGCCCGTGACCTCACTACTGATATTATCTGTTTCAATAACGATGTTCACTTGGATGCTAGTGGTAGCAGAATTTTGAACCTAGCGAACGCCGTGGATGACCGCGCTTCTGTACCGTTTGGTCAACTAAAGACCATGGTCCAACACTTATGGCATGCACTGAATCAATCCTTACATCTCCCTATTGAgtacgagac                                                                                                                                                                                                                                                   >  1:337408/1‑293 (MQ=255)`  
                                                        `aagacagcTCCACTACCGTCCGATTGGTTGACTTTACGGATTGTTCAATCCTCCGCGCGGATGACTTTAACGTCGCTCAGATTCAAACGATGCACGTAGCGGAAGAGGCCCGTGACCTCACTACGGATATTAGCGGTGGCAATAACGATGGTCACTTGGATGCTCGTGGGCGTCGAATTGTGAACATAGCGAACGCCGTGGATGACCGCGATGCTGTTCCGTTTGGTCAACTAAAGACCATGGACCAGAACTCATGGCAAGCACGTAATGAAGCCTTACAGTTCCGTAATGAGGCTGAGAc                                                                                                                                                                                                                                                   <  1:217481/294‑1 (MQ=255)`  
                                                              `ccTCCACTACCGACCGATTGGTTAACTTTACGGATGGTTCAATCCTCCGCGCGTATGACCTTAACGTCGCTCAGATTCAAACGATGCACGTAGCGGAAGAGGCCCGTGACCTCACTACGGATATTATCGGTGTCAATAACGATGGTCACTTGGATGCTCGTGGTCGTCGAATTGTGAACCTAGCGAACGCCGTGGATGACCGCGATGCTGTTCCGTTTGGTCAACTAAAGACCATGGACCAGAACTCATGGCAAGCACGTAATGAAGCCTTAcctgtctcttatacacatctccgagccca                                                                                                                                                                                                                                             >  1:508269/1‑273 (MQ=255)`  
                                                               `cTCCACTACCGACCGATTGGTTGACTTTACGGATGGTTCAATCCTCCGCGCGTATGACCTTAACGTCGCTCAGATTCAAACGATGCACGTAGCGGAAGAGGCCCGTGACCTCACTACGGATATTATCGGTGTCAATAACGATGGTCACTTGGATGCTCGTGGTCGTCGAATTGTGAACCTAGCGAACGCCGTGGATGACCGCGATGCTGTTCCGTTTGGTCAACTAAAGACCATGGACCAGAACTCATGGCAAGCACGTAATGAAGCCTTACAGTTCCGTaactgtctcttataaacctcc                                                                                                                                                                                                                                            >  1:277938/1‑282 (MQ=255)`  
                                                               `cTCCACTACCGACCGATTGGTTAACTTTACGGATGGTTCAATCCTCCTCGCGTATGACCTTAACGTCGCTCAGATTCAAACGATGCACGTAGCGGAAGAGGCCCGTGACCTCACTACGGATATTATCGGTGTCAATAACGATGGTCACTTGGATGCTCGTGGTCGTCGAATTGTGAACCTAGCGAACGCCGTGGATGACCGCGATGCTGTTCCGTTTGGTCAACTAAAGACCATTGACCAGAACTCATGGCAAGCACGTAATGAAGCCTTACAGTTCCGTAATGAGGCTGAGACTTTCAGa                                                                                                                                                                                                                                            >  1:264559/1‑301 (MQ=255)`  
                                                                           `ccgaTTGGTTGACTTTACGGACGGTTCAATCCTCCGCGCGTATGACCTTAACGTCGCTCAGATTAAAACGATGCACGTAGCGGAAGAGGCCCGTGACCTCACTACGGATATTATCGGTGTCAATAACGATGGTCACTTGGATGCTCGTGGTCGTCGAATTGTGAACCTAGCGAACGCCGTGGATGACCGCGATGCTGTTCCGTTTGGTCAACTAAAGACCATGGACCAGAACTCATGGCAAGCACGTAATGAAGCCTTACAGTTCCGTAATGAGGCTGAGACTTTCAGAAACCAAGCGGAg                                                                                                                                                                                                                                <  1:367955/301‑1 (MQ=255)`  
                                                                            `cgaTTGGTTGACTTTACGGATGGTTCAATCCTCCGCGCGTATGACCTTAACGTCTCTCCGTTTCAAACGATTCTCCTTGCGCAAGTTTCCCTTTTCCTCTCTTCTTCTCTTATCCCTTTCATTAACGTTTCTCTCTTGTTTTCTCGTTGTCGTCGTATTTTTATCCTAGCGCTCTCCGTTGATCCCCCCTATGCTGTTCCGTTTGGTCAACTAAAGTCCATGGACCTGTACTCATGGCAAGCACGTAATGACGCCTTACAGTTCCGTAATGAGCCTTAGACTTTCCGCAAccctgtctctt                                                                                                                                                                                                                               >  1:14/1‑292 (MQ=255)`  
                                                                                   `ttaaCTTTACGGATGGTTAAATCCTCCGCGCGTATGACCTTAACGTCGCTCAGATTCAAACGTTGCACGGAGCGGAAGAGGCCCGTGACCTCACTACGGAGATTAGCGGTGTCAATAACGATGGTCACTTGGATGCTCGTGGTCGTCGAATTGTGAACCTAGCGAACGCCGTGGATGACCGCGATGCTGTTCCGTTTGGTCAACTAAAGACCATGGACCAGAACTCATGGCAAGCACGTAATGAAGCCTTACAGTTCCGTAATGAGGCTGAGACTTTCAGAAACCAAGCGGAGGGCTTTaa                                                                                                                                                                                                                        <  1:259067/298‑1 (MQ=255)`  
                                                                                     `tgtgtataagaaacagcAATCCTCCGCGCGTATGACCTTAACGTCGCTCAGATTCAAACGATGCACGTAGCGGAAGAGGCCCGTGACCTCACTACGGATATTATCGGTGTCAATAACGATGGTCACTTGGATGCTCGTGGTCGTCGAATTGTGAACCTAGCGAACGCCGTGGATGACCGCGATGCTGTTCCGTTTGGTCAACTAAAGACCATGGACCAGAACTCATGGCAAGCACGTAATGAAGCCTTACAGTTCCGTAATGAGGCTGAGACTTTCAGAAACCAAGCGGAGGGCTTTAAGa                                                                                                                                                                                                                      <  1:1368/285‑1 (MQ=255)`  
                                                                                      `aCTTTACGGATGGTTCAATCCTCCGCGCGTATGACCTTAACGTCGCTCAGATTCAAACGATGCACGTAGCGGAAGAGGCCCGTGACCTCACTACGGATATTATCGGTGTCAATAACGATGGTCACTTGGATGCTCGTGGTCGTCGAATTGTGAACCTAGCGAACGCCGTGGATGACCGCGATGCTGTTCCGTTTGGTCAACTAAAGACCATGGACCAGAACTCATGGCAAGCACGTAATGAAGCCTTACAGTTCCGTAATGAGGCTGAGACTTTCAGAAACCAAGCGGAGGGCTTTAAGaa                                                                                                                                                                                                                     <  1:131513/301‑1 (MQ=255)`  
                                                                                       `cTTTACGGATGGTTCAATCCTCCGCGCGTATGACCTTAACGTCGCTCAGATTCAAACGATGCACGTAGCGGAAGAGGCCCGTGACCTCACTACGTATACTATCGGTGTCAATAACGATGGTCACTTGGATGCTCGTGGTCGTCGAATTGTGAACCTAGCGAACGCCGTGGATGACCGCGCTGCTGTTCCGTTTGGTCACCTAAAGACCATGGACCAGACCTCATGGCAAGCACGTAATGAAGCCTTACCGTTCCGTAATGAGGCTGAGCCTTTCAGAAACCAAGCCGAGGGCTTTAAgcag                                                                                                                                                                                                                    >  1:433357/1‑298 (MQ=255)`  
                                                                                                `tGGTTCAATCCTCCGCGCGTATGACCTTAACGTCGCTCAGATTCAAACGATGCACGTAGCGGAAGAGGCCCGTGACCTCACTACGGATATTATCGGTGTCAATAACGATGGTCACTTGGATGCTCGTGGTCGTCGAATTGTGAACCTAGCGAACGCCGTGGATGACCGCGATGCTGTTCCGTTTGGTCAACTAAAGACCATGGACCAGAACTCATGGCAAGCACGTAATGAAGCCTTACAGTTCCGTAATGAGGCTGAGACTTTCAGAAACCAAGCgctgtctcttatacacatctgacgc                                                                                                                                                                                                           <  1:299899/301‑25 (MQ=255)`  
                                                                                                  `gTTCAATCCTCCGCGCGTATGACCTTAACGTCGCTCAGATTCAAACGATGCACGTAGCGGAAGAGGCCCGTGACCTCACTACGGATATTATCGGTGTCAATAACGATGGTCACTTGGATGCTCGTGGTCGTCGAATTGTGAACCTAGCGAACGCCGTGGATGACCGCGATGCTGTTCCGTTTGGTCAACTAAAGACCATGGACCAGAACTCATGGCAAGCACGTAATGAAGCCTTACAGTTCCGTAATGAGGCTGAGACTTTCAGAAACCCACCGGAGGGCTTTAAGAActgtctcttata                                                                                                                                                                                                         >  1:318983/1‑290 (MQ=255)`  
                                                                                                  `gTTCAATCCTCCGCGCGTATGACCTTAACGTCGCTCAGATTCAAACGATGCACGTAGCGGAAGAGGCCCGTGACCTCACTACGGATATTATCGGTGTCAATAACGATGGTCACTTGGATGCTCGTGGTCGTCGAATTGTGAACCTAGCGAACGCCGTGGATGACCGCGATGCTGTTCCGTTTGGTCAACTAAAGACCATGGACCAGAACTCATGGCAAGCACGTAATGAAGCCTTACAGTTCCGTAATGAGGCTGAGACTTTCAGAAACCAAGCGGAGGGATTTAAGAACGAGTCCAGTAc                                                                                                                                                                                                         >  1:91043/1‑301 (MQ=255)`  
                                                                                                  `gTTCAATCCTCCGCGCGTATGACCTTAACGTCGCTCAGATTCAAACGATGCACGTAGCGGAAGAGGCCCGTGACCTCACTACGGATATTATCGGTGTCAATAACGATGGTCACTTGGATGATCGTGGTCGTCGAATTGTGAACCTAGCGAACGCCGTGGATGACCGCGATGCTGTTCCGTTTGGTCAACTAAAGACCATGGACCAGAACTCATGGCAAGCACGTAATGAAGCCTTACAGTTCCGTAATGAGGCTGAGACTTTCAGCAACCAAGCGGAGGGCTTTAAGAACGAGTCCAGTAc                                                                                                                                                                                                         >  1:171229/1‑301 (MQ=255)`  
                                                                                                  `gTTCAATCCTCCGCGAGTATGACCTTAACGTCGCTCAGATTCAAACGATGCACGTAGCGGAAGAGGCCCGTGACCTCACTACGGATATTATCGGTGTCAATAACGATGGTCACTTGGATGCTCGTGGTCGTCGAATTGTGAACCTAGCGAACGCCGTGGATGACCGCGATGCTGTTCCGTTTGGTCAACTAAAGACCATGGACCAGAACTCATGGCAAGCACGTAATGAAGCCTTACAGTTCCGTAATTAGGCTGAGACTTTCAGAAACCAAGCGGAGGGCTTTAAGAACGAGTCCAGTAc                                                                                                                                                                                                         >  1:486574/1‑301 (MQ=255)`  
                                                                                                   `ttCAATCCTCCTCTCGTATTACCTTCACGCCCCTCATATTCAACCGATGCCCGTTGCGGAAGAGGCCCGTTCCCTCCCTACGGATATTATCGGTCTCAATAACGATGGTCACTTGGATGCTCGTGGTCGTCGAATTGTGAACCTAGCGAACTCCGTGGATGACCGCGATGCTGTTCCGTTTAGTCAACTAAAGACCATGGACCAGAACTCATGGCAAGCACGTAATGAAGCCTTACAGTTCCGTAATGAGGCTGAGACTTTCAGAAACCAAGCGGAGGGCTTTAAGAACGAGTCCAGTAcc                                                                                                                                                                                                        <  1:353835/301‑1 (MQ=255)`  
                                                                                                           `tccGCGCGTATGACCTTAACGTCGCTCAGATTCAAACGATGCACGTAGCGGAAGAGGCCCGTGACCTCACTACGTATACTATCGGTGTCAATAACGATGGTCACTTGGATGCTCGTGGTCGTCGAATTGTGAACCTAGCGAACGCCGTGGATGACCGCGATGCTGTTCCGTTTGGTCAACTAAAGACCCTGGACCAGAACTCATGGCAAGCACGTAATGAAGCCTTACAGTTACGTAATGAGGCTGATACTTTCAGAAACCAAGCGGAGGGCTTTAAGAACGAGTCCAGTACCAACGCTAc                                                                                                                                                                                                >  1:178548/1‑301 (MQ=255)`  
                                                                                                                 `gagacagccTTAACGTCGTTCAGATTCAAACGATGCACGTAGCGGAAGAGGCCCGTGACCTCACTACGGATATTATCGGTGTCAATAACGATGGTCACTTGGATGCTCGTGGTCGTCGAATTGTGAACCTAGCTAACGCCGTGGATGACCGCGATGCTGTTCCGTTTGGTCAACTAAAGACCATGGACCAGAACTCATGGCAAGCACGTAATGAAGCCTTACAGTTCCGTAATGAGGCTGAGACTTTCAGAAACCAAGCGGAGGGCTTTAATAACGAGTCCAGTACCAACGCTACGAacac                                                                                                                                                                                          <  1:76670/294‑1 (MQ=255)`  
                                                                                                                 `cggagatgtgtataagagacaggATTCAAACGATGCACGTAGCGGAAGAGGCCCGTGACCTCACTACGGATATTATCGGTGTCAATAACGATGGTCACTTGGATGCTCGTGGTCGTCGAATTGTGAACCTAGCGAACGCCGTGGATGACCGCGATGCTGTTCCGTTTGGTCAACTAAAGACCATGGACCAGAACTCATGGCAAGCACGTAATGAAGCCTTACAGTTCCGTAATGAGGCTGAGACTTTCAGAAACCAAGCGGAGGGCTTTAAGAACGAGTCCAGTACCAACGCTACGAacac                                                                                                                                                                                          <  1:231490/279‑1 (MQ=255)`  
                                                                                                                 `cgTATGACCTTAACGTCGCTCAGATTCAAACGAGGCACGTAGCGGAAGAGGCCCGTGACCTCACTACGGATATTATCGGTGTCAATAACGATGGTCACTTGGATGCTCGTGGTCGTCGAATTGTGAACCTAGCGAACGCCGTGGATGACCGCGATGCTGTTCCGTTTGGTCAACTAAAGACCATGGACCAGAACTCATGGCAAGCACGTAATGAAGCCTTACAGTTCCGTAATGAGGCTGAGACTTTCAGAAACCAAGCGGAGGGCTTTAAGAACGAGTCCAGTACCAACGCTACGAacac                                                                                                                                                                                          <  1:458527/301‑1 (MQ=255)`  
                                                                                                                      `gcccTTAACGTCGCGCATATTCAAAGGATGCCCGTAGCGGAAGAGGCCCGTGACCGCACTACGGATATTATCGGTGTCAATAACGATGGTCACTTGGATGCTCGTGGTCGTCGAATTGTGAACCTAGCGAACGCCGTGGATGACCGCGATGCTGTTCCGTTTGGTCAACTAAAGACCATGGACCAGAACTCATGGCAAGCACGTAATGAAGCCTTACAGTTCCGTAATGAGGCTGAGACTTTCAGAAACCAAGCGGAGGGCTTTAAGAACGAGTCCAGTACCAACGCTACGAACACAAAGc                                                                                                                                                                                     <  1:64723/299‑1 (MQ=255)`  
                                                                                                                        `ccTTAACGTCGCTCAGATTCAAACGATGCACGTAGCGGAAGAGGCCCGTGACCTCACTACGGATATTATCGGTGTCAATAACGATGGTCACTTGGATGCTCGTGGTCGTCGAATTGTGAACCTAGCGAACGCCGTGGATGACCGCGATGCTGTTCCGTTTGGTCAACTAAAGACCATGGACCAGAACTCATGGCAAGCACGTAATGAAGCCTTACAGTTCCGGAATGATGCTGAGACTTTCAGAAACCAAGCGGAGGGCTTTAAGAACGAGTCCAGTACCACCGCTACGAACACAAAGCAg                                                                                                                                                                                   >  1:380430/1‑301 (MQ=255)`  
                                                                                                                                    `ccaagttCAATCGATTGCCGTATCGGAAGAGTCCCGTGACCTCACTACTGATATTATCGGTTTCAATAACGCTGGTTACTTGGATTCTCGTGGTCGTCGAATTGTGAACCTAGCGAACGCCGTGGATGACCGCGATGCTGTTCCGTTTGGTCAACTAAAGACCATGGACCAGAACTCATGGCAAGCACGTAATGAAGCCTTACAGTTCCGTAATGAGGCTGAGACTTTCAGAAACCAAGCGGAGGGCTTTAAGAACGAGTCCAGTACCAACGCTACGAACACAAAGCAGTGGCGCGATgat                                                                                                                                                                       <  1:111571/296‑2 (MQ=255)`  
                                                                                                                                       `gATTCAAACGATGCACGTAGCGGAAGAGGCCCGTGACCTCACTACGGATATTATCGGTGTCAATAACGATGGTCACTTGGATGCTCGTGGTCGTCGAATTGTGAACCTAGCGAACGCCGTGGATGACCGCGATGCTGTTCCGTTTGGTCAACTAAAGACCATGGACCAGAACTCATGGCAAGCACGTAATGAAGCCTTACAGTTCCGTAATGAGGCTGAGAATTTCAGAAACCAAGCGGAGGGCTTTAAGAACGAGTCCAGTACCAACGATACGAACACAAAGCAGTGGCGCGATGAGAcc                                                                                                                                                                    >  1:385367/1‑301 (MQ=255)`  
                                                                                                                                            `aaaCGATGCCCGTAGCGGAAGAGGCCCGTGACCTCACTACGGATATTATCGGTGTCAATAACGATGGTCACTTGGATGCTCGTTGTCGTCGAATTGTGAACCTAGCGAACGCCGTGGATGACCGCGATGCTGTTCCGTTTGGTCAACTAAAGACCATGGACCAGAACTCATGGCAAGCACGTAATGAAGCCTTACAGTTCCGTAATGAGGCTGAGACTTTCAGAAACCAAGCGGAGGGCTTTAAGAACGAGTCCAGTACCAACGCTACGAACACAAAGCAGTGGCGCGATGAGACCAAggg                                                                                                                                                               <  1:409493/301‑1 (MQ=255)`  
                                                                                                                                                  `agacagTAGCGGAAGAGGCCCGTGACCTCACTACGGATATTATCGGTGTCAATAACGATGGTCACTTGGATGCTCGTGGTCGTCGAATTGTGAACCTAGCGAACGCCGTGGATGACCGCGATGCTGTTCCGTTTGGTCAACTAAAGACCATGGACCAGAACTCATGGCAAGCACGTAATGAAGCCTTACAGTTCCGTAATGAGGCTGAGACTTTAAGAAACCAAGCGGAGGGCTTTAAGAACGAGTCCAGTACCAACGCTACGAACACAAAGCAGTGGCGCGATGAGACCAAGGGTTTCCg                                                                                                                                                         <  1:56530/296‑1 (MQ=255)`  
                                                                                                                                                          `gCGGAAGAGGCCCGTGACCTCACTACGGATATTATCGGTGTCAATAACGATGGTCACTTGGATGCTCGTGGTCGTCGAATTGTGAACCTAGCGAACGCCGTGGATGACCGCGATGCTGTTCCGTTTGGTCAACTAAAGACCATGGACCAGAACTCATGGCAAGCAAGTAATGAAGCCTTACAGTTCCGTAATGAGGCTGAGAATTTCAGAACCCAAGCGGAGGGCTTTAAGAACGACTCACGTCACCACGCTACGAACAGAAAGCAGTGGCGCGATTAGACCAAAGGTTTCCGAGaagaag                                                                                                                                                 >  1:245565/1‑301 (MQ=255)`  
                                                                                                                                                          `gCGGAAGAGGCCCGTGACCTCACTACGGATATTATCGGTGTCAATAACGATGGTCACGTGGATGCTCGTGGTCGTCGAATTTTGAACCTAGCGAACGCCGTGGATGACCTCGATGCGGTTCCGTTTGGTCAACTAAAGACCATGGACCAGAACTCATGGCAGGCACGTAATGAAGCCTGACAGTTCCGTAATGATGCGGAGACTTGCAGATACCAAGCGGAGAGCGTTAAGAACGAGTCCAGTACCAACGCAAGGAACACAAGACAGTGGCGCGATGAGACCAAGGGTAGCCGGGACGAAg                                                                                                                                                 >  1:96007/1‑301 (MQ=255)`  
                                                                                                                                                               `agagcaccGTGACCTAACGACTGATATTATCGGTGTCAATAACGATGGTCATTTGGAGGTTCGTGGGCGTCGAATTGTGAACCTGGCGAACGCCGGGTATGACCGCGATGCTGTTCCGTTTGGTCAACTAAAGACCATGGACCAGAACTCATGGCAAGCACGTAATGAAGCCTTACAGTTCCGTAATGAGGCTGAGACTTTCAGAAACCAAGCGGAGGGCTTTAAGAACGAGTCCAGTACCAACGCTACGAACACAAAGCAGTGGCGCGATGAGACCAATGGTTTCCGAGACGAAGCCGAg                                                                                                                                            <  1:42318/295‑1 (MQ=255)`  
                                                                                                                                                                `gagGCCCGTGACCTCACTACGGATATTATCGGTGTCAATAACGATGGTCACTTGGATGCTCGTGGTCGTCGAATTGTGAACCTAGCGAACGCCGTGGATGACCGCGATGCTGTTCCGTTTGGTCAACTAAAGACCATGGACCAGAACTCATGGCAAGCACGTAATGAAGCCTTACAGTTCCGTAATGAGGCTGAGACTTTCAGAAACCAAGCGGATGGCTTTAAGAACGAGTCCAGTACCAACGCTACGAACACAAAGCAGTGGCGCGATGAGACCAAGGGTTTCCGAGACGAAGCCGAGc                                                                                                                                           <  1:504061/301‑1 (MQ=255)`  
                                                                                                                                                                          `cgtccccatgggATATTAGCGCTGTCAATCACCATGGTCGCTTGGATGCTAGTGTGCGTCGAATTGTGACCCTCGCGAACGCCGTGGATTACCTCGATGCTGTTCCGTTTGGTCAACTAAAGACCATGGAACAGAACTCATGGCAAGCACGTAATGAAGCCTTACAGTTCCGTAATGAGGCTGAGACTTTCAGAAACCAAGCGGAGGGCTTTAAGAACGAGTACAGTACCAACGCTATGAACACAAAGCAGTGGCGCGATGAGACCAAGGGTTTCCGAGACGAAGCCAAGCGGTTCAAGaa                                                                                                                                 <  1:491364/291‑1 (MQ=255)`  
                                                                                                                                                                          `ataagagacaggATATTAGCGGTGTCAATAACGATGGTCACTTGGATGCTCGTGGTCGTCGAATTGTGAACCTAGCGAACGCCGTGGATGACCGCGATGCTGTTCCGTTTGGTCAACTAAAGACCATGGACCAGAACTCATGGCAAGCACGTAATGAAGCCTTACAGTTCCGTAATGAGGCTGAGACTTTCAGAAACCAAGCGGAGGGCTTTAAGAACGAGTCCAGTACCAACGCTACGAACACAAAGCAGTGGCGCGATGAGACCAAGGGTTTCCGAGACAAAGCCGAGCGGTTCAAGaa                                                                                                                                 <  1:453829/291‑1 (MQ=255)`  
                                                                                                                                                                           `ccTCACTACGGATATTATCGGTGTCAATAACGATGGTCACTTGGATGCTCGTGGTCGTCGAATTGTGAACCTAGCGAACGCCGTTGATGACCGCGATGCTGTTCCGTTTGGTCAACTAAAGACCATGGACCAGAACTCATGGCAAGCACGTAATGAAGCCTTACAGTTCCGTAATGAGGCTGAGACTTTCAGAAACCAAGCGGAGGGCATTAAGAACGAGTCAAGTACAAATGCTACGAACAAAAAGCAGTGGCGCGATGAGACCAAAGGGTTCCGAAACGAAGCCAAGCGGGTCacgtaa                                                                                                                                >  1:226464/1‑296 (MQ=255)`  
                                                                                                                                                                            `actgcTACGTATACAATCGGTGTCAATAACGATGGTCACTTGGATGCTCGTGGTCGTCGAATTGTGAACCTAGCGAACGCCGTGGATGACCGCGATGCTGTGCCGTTTGGTCAACTAAAGACCATGGACCAGAACTCATGGCAAGCACGTAATGAAGCCTTACAGTTCCGTAATGAGGCTGAGACTTTCAG‑AACCAAGCGGAGGGCTTTAAGAACGAGTCCAGTACCAACGCTACGAACACAAAGCAGTGGCGCGATGAGACCAAGGGTTTCCGAGACGAAGCCAAGCGGTTCAAGAATAc                                                                                                                              <  1:296285/297‑1 (MQ=255)`  
                                                                                                                                                                              `cACTACGGATATTATCGGTGTCAATAACGATGGTCACTTGGATGCTCGTGGTCGTCGAATTGTGAACCTAGCGAACGCCGTGGATGACCGCGATGCTGTTCCGTTTGGTCAACTAAAGACCATGGACCAGAACTCATGGCAAGCACGTAATGAAGCCTTACAGTTCCGTAATGAGGCTGAGACTTTCAGAAACCAAGCGGAGGGCTTTAAGAACGAGTCCAGTACCAACGCTACGAACACAAAGCAGTGGCGCGATGAGACCAAGGGTTTCCGAGACGAAGCCAAGCGGTTCAAGAATACg                                                                                                                             >  1:162233/1‑301 (MQ=255)`  
                                                                                                                                                                                `ctccGGATATTATCGGTGTCAATAACGATGGTCACTTGGATGCTCGTTGTCGTCGACTTGTGAACCTAGCGAACGACGTGGATGACCGCGATGCTGTTCCGTTTGGTCAACTAAAGACCATTGACCAGAACTCATGGCAAGCACCTAATAGAGCCTTACAGTTCCGTAATGAGGCTGAGACTTTCGGAAACCAAGCGGAGGCCTTTAAGAACTAGTCCAGTACCAACGCTACTAACACAAAGCAGTTGCGCGATGCGACCACGAGTTTCCGAGACGAAGCCGAGCGCTTACAGaactgtct                                                                                                                           >  1:359314/4‑295 (MQ=255)`  
                                                                                                                                                                                `cTACGGATATTATCGTTGTCAATAACGATGGTCACTTGGATGTGCGTGGTCGTCGAATTGTTAACCTAGCGAACCCCGTGGATGACCGCGATGCTGTTCCGTTTGGTCAACTAAAGACCATGGACCAGAACTCATGGCAAGCACGTAATGAAGCCTTACAGTTCCGTAATGAGGCTGAGACTTTCAGAAACCAAGCTGAGGGCTTTAAGAACGAGTCCAGTACCAACGCTACGAACACAAAGCAGTGGCGCGATGAGACCAATGGTTTCCGAGACGAAGCCAAGCGGTTAAAGAATACGGc                                                                                                                           <  1:192134/301‑1 (MQ=255)`  
                                                                                                                                                                                `cTACGGATATTATCGGTGTCAATAACGATGGTCACTTGGATGCTCGTGGTCGTCGAATTGTGAACCTAGCGAACGCCGTGGATGACCGCGATGCTGTTCCGTTTGGTCAACTAAAGACCATGGACCAGAACTTATGGCAAGCACGTAATGAAGCCTTACAGTTCCGTAATGAGGCTGAGACTTTCAGAAACCAAGCGGAGGGCTTTAAGAACGAGTCCAGTACCAACGCTACGAACACAAAGCAGTGGCGCGATTAGACCAAGGGTTTCCGAGACGAAGCCAAGCGGTTCAAGaactgtct                                                                                                                           >  1:374678/1‑295 (MQ=255)`  
                                                                                                                                                                                `agatgggtataagagacagcAATAACGATGGTCACTTGGATGCTCGTGGTCGTCGAATTGTGAACCTAGCGAACGCCGTGGATGACCGCGATGCTGTTCCGTTTGGTCAACTAAAGACCATGGACCAGAACTCATGGCAAGCACGTAATGAAGCCTTACAGTTCCGTAATGAGGCTGAGACTTTCAGAAACCAAGCGGAGGGCTTTAAGAACGAGTCCAGTACCAACGCTACGAACACAAAGCAGTGGCGCGATGAGACCAAGGGTTTCCGAGACGAAGCCGAGCGGTTCAAGAATACGGc                                                                                                                           <  1:406103/282‑1 (MQ=255)`  
                                                                                                                                                                                     `gATATTATCGGTGTCAATAACGATGGTTACTTGGATGCTCGTGGTCGTCGAATTGTGAACCTAGCGAACGCCGTGGATGACCGCGATGCTGTTCCGTTTGGTCAACTAAAGACCATGGACCAGAACTCATGGCAAGCACGTAATGAAGCCTTACAGTTCCGTAATGAGGCTGAGACTTTCAGAAACCAAGCGGAGGGCTTTAAGAACGAGTCCAGTACCAACGCTACGAACACAAAGCAGTGGCGAGATGAGACCAAGGGTTTCCGAGACGAAGCCAAGCGGTTCAAGAATATGGCTGGTc                                                                                                                      <  1:471458/301‑1 (MQ=255)`  
                                                                                                                                                                                      `atatTATCGGTGTCAATAACGATGGTCACTTGGATGCTCGTGGTCGTCGAATTGTGAACCTAGCGAACGCCGTGGATGACCGCGATGCTGTTCCGTTTGGTCAACTAAAGACCATGGACCAGAACTCATGGCAAGCACGTAATGAAGCCTTACAGTTCCGTAATGAGGCTGAGACTTTCAGAAACCAAGCGGAGGGCTTTAAGAACGAGTCAAGTACCAACGCTACGAACACAAAGCAGTGGCGCGATGAGACCAAGGGTTTCCGAGACGAAGCCGAGCGGTTCAAGaactgtctcttata                                                                                                                     >  1:273733/1‑289 (MQ=255)`  
                                                                                                                                                                                           `aTCGGTGTCAATAACGATGGTCACTTGGATGCTCGTGGTCATCGAATTGTGAACCTAGCGAACGCCGTGGATGACCGCGATGCTGTTCCGTTTGGTCAACTAAAGACCATGGACCAGAACTCATGGCAAGCACGTAATGAAGCCTTACAGTTCCGTAATGAGGCTGAGACTTTCAGAAACCAAGCGGAGGGCTTTAAGAACGAGTCCAGTACCAACGCTACGAACACAAAGCAGTGGCGCGATGAGACCAAGGGTTTCCGAGACGAAGCCAAGCGGTTCGAGGATACGGCTGGTCAATACg                                                                                                                >  1:18262/1‑301 (MQ=255)`  
                                                                                                                                                                                               `gtgtCAATAACGATGGTCACTTGGATGCTCGTGGTCGTCGAATTGTGAACCTAGCGAACGCCGTGGATGACCGCGATGCTGTTCCGTTTGGTCAACTAAAGACCATGGACCAGAACTCATGGCAAGCACGTAATGAAGCCTTACAGTTCCGTAATGAGGCTGAAACTTTCAGAAACCAAGCGGAGGGCCTGAAGAACGAGTCCAGTACACACGCTTCGAACACAAAGCAGTGGCGCTATGAGACCAAGGGTTTCCGCGACGAAGCCGAGCGGAGCAAGCATAAGGCTGGTCCATAAGCTaa                                                                                                            >  1:418693/1‑300 (MQ=255)`  
                                                                                                                                                                                                `tgtCAATAACGATGGTCACTTGGATGCTCGTGGTCGTCGAATTGTGAACCTAGCGAACGCCGTGGTTGACCGCGATGCTGTTCCGTTTGGTCAACTAAAGACCATGGACCAGAACTCATGGCAAGCACGTAATGAAGCATTACAGTTCCGTAATGAGGCTGAGACTTTCAGAAACCAAGCGGAGGGCTTTAAGAACGAGTCCAGTACCAACGCTACGAACACAAAGCAGTGGCGCGATGAGACCAAGGGTTTCCGAGACGAAGCCAAGCGGTTCAAGAATACGGCTGGTCAATACGCTACa                                                                                                           >  1:236863/1‑301 (MQ=255)`  
                                                                                                                                                                                                `tgtCAATAACGATGGTCACTTGGATGCTCGTGGTCGTCGAATTGTGAACCTAGCGAACACCGTGGATGACCGCGATGCTGTTCCGTTTGGTCAACTAAAGACCATGGACCAGAACTCATGGCAAGCACGTAATGAAGCCTTACAGTTCCGTAATGAGGCTGAGACTTTCAGAAACCAAGCGGAGGGCTTTAAGAACGAGTCCAGTACCAACGCTACGAACACAAAGCAGTGGCGCGATGAGACCAAGGGTTTCCGAGACGAAGCCAAGCGGTTCAAGAATACGGCTGGTCAATACGCTACa                                                                                                           >  1:439211/1‑301 (MQ=255)`  
                                                                                                                                                                                                  `tCAATAACGATGGTCACTTGGATGCTCGTGGTCGTCGAATTGTGAACCTAGCGAACGCCGTGGATGACCGCGATGCTGTTCCGTTTGGTCAACTAAAGACCATGGACCAGAACTCATGGCAAGCACGTAATGAAGCCTTACAGTTCCGTAATGAGGCTGAGACTTTCAGAAACCAAGCGGAGGGCTTTAAGAACGAGTCCAGTACCAACGCTACGAACACAAAGCAGTGGCGCGATGAGACCAAGGGTTTCCGAGACGAAGCCGAGCGGTTCAAGAATACGGCTGGTCAATACGCTACATc                                                                                                         <  1:100000/301‑1 (MQ=255)`  
                                                                                                                                                                                                   `cAATAACGATGGTCACTTGGATGCTCGTGGTCGTCGAATTGTGAACCTAGCGAACGCCGTGGATGACCGCGATGCTGTTCCGTTTGGTCAACTAAAGACCATGGACCAGAACTCATGGCAAGCACGTAATGAAGCCTTACAGTTCCGTAATGAGGCTGAGACTTTCAGAAACCAAGCGGAGGGCTTTAAGAACGAGTCCAGTACCAACGCTACGAACACAAAGCAGTGGCGCTATGAGACCAAGGGTTTCCGAGACGAAGCCAAGCGGTTCAAGAATACGGCTGGTCAATAcctgtctctt                                                                                                        >  1:268951/1‑292 (MQ=255)`  
                                                                                                                                                                                                   `cAATAACGATGGTCACTTGGATGCTCGTGGTCGTCGAATTGTGAACCTAGCGAACGCCGTGGATGACCGCGATGCTGTTCCGTTTGGTCAACTAAAGACCATGGACCAGAACTCATGGCAAGCACGTAATGAAGCCTTACAGTTCCGTAATGAGGCTGAGACTTTCAGAAACCAAGCGGAGGGCTTTAAGAACGAGTCCAGTACCAACGCTACGAACACAAAGCAGTGGCGCGATGAGACCAAGGGTTTCCGAGACGAAGCCAAGCGGTTCAAGAATACGGCTGGTCAATACGCTACATct                                                                                                        >  1:166408/1‑301 (MQ=255)`  
                                                                                                                                                                                                            `agacagctgggATGCTCGTGGTCGTTGAATTGTGAACCTAGCGAACGCCGTGGAGGAACGAGGTGCTTTTCCGGTTGGTCAACTAAAGACCATGGACCAGAACTCATGGCAAGAACGTAATGAAGCCTTACAGTTCCGTAATGAGGCTGAGACTTTCAGTAACCAAGAGGAGGGCTTTAAGAACGAGTCCAGTACCAACGCTACGAACACAAAGCAGTGGCGCGATGAGACCAAGGGTTGCCGAGACGAAGCGAAGCAGTTCAAGAATACGGCTGGTCAATACGCTACATCTGCTGGGAAc                                                                                               <  1:210163/292‑1 (MQ=255)`  
                                                                                                                                                                                                             `ggTCACTTGGATGCTCGTGGTCGTCGAATTGTGAACCTAGCGAACGCCGTGGATGACCGCGATGCTGTTCCGTTTGGTCAACTAAAGACCATGGACCAGAACTCATGGCAAGCACGGAATGAAGCCTTACAGTTCCGTAATGAGGCTGAGACTTTCAGAAACCAAGCGGAGGGCTTTAATAACGAGTCCAGTACCAACGCTACGAACACAAAGCAGTGGCGCGATGAGACCAAGGGGTTCCGAGACGAAGCCAAGCGGTTCAAGAATACGGCTGGTCAATAcaacacctgactcttataaa                                                                                              >  1:480580/1‑282 (MQ=255)`  
                                                                                                                                                                                                              `gTCACTTGGATGCTCGTGGTCGTCGAATTGTGAACCTAGCGAACGCCGTGGATGACCGCGATGCTGTTCCGTTTGGTCAACTAAAGACCATGGACCAGAACCCATGGCAAGCACGTAATGAAGCCTTACAGTTCCGTAATGAGGCTGAGACTTTCAGAAACCAAGCGGAGGGCTTTAAGAACGAGTCCAGTACCAACGCTACGAACACAAAGCAGTGGCGTGATGAGACCAAGGGTTTCCGAGACGAAGCCGAGCGGTTCAAGAATACGGATGGTCAATACGCTACATCTGCTGGGAActc                                                                                             >  1:394598/1‑301 (MQ=255)`  
                                                                                                                                                                                                                `cACTTGGATGCTCGTGGTCGTCGAATTGTGAACCTAGCGAACGCCGTGGATGACCGCGATGCTGTTCCGTTTGGTCAACTAAAGACCATGGACCAGAACTCATGGCAAGCACGTAATGAAGCCTTACAGTTCCGTAATGAGGCTGAGACTTTCAGAAACCAAGCGGAGGGCTTTAAGAACGAGTCCAGTACCAACGCTACGAACACAAAGCAGTGGCGCGATGAGACCAAGGGTTTCCGAGACGAAGCCAAGAGGTTAAAGAATATGGCTGGTCAATACGCTACATCTGCTGGGAActcct                                                                                           >  1:495870/1‑299 (MQ=255)`  
                                                                                                                                                                                                                     `ggATGCTCGTGGTCGTCGAATTGTGAACCTAGCGAACGCCGTGGATGACCGCGATGCTGTTCCGTTTGGTCAACTAAAGACCATGGACCAGAACTCATGGCAAGCACGTAATGAAGCCTTACAGTTCCGTAATGAGGCTGAGACTTTCAGAAACCAAGCGGAGGGCTTTAAGAACGAGTCCAGTACCAACGCTACGAACACAAAGCAGTGGCGCGATGAGACCAAGGGTTTCCGAGACGAAGCCGAGCGGTTCAAGAATACGGCTGGTCAATACGCTACATCTGCTGGGAACTCTGCTTcc                                                                                      <  1:475152/301‑1 (MQ=255)`  
                                                                                                                                                                                                                                   `agagacaggtgAACATAGCGAACGCCGGGGAATACCGGGATGCTGTTCCGGTTGGTCAACTAAAGACCATGGACCAGAACTAATGGCAAGCACGTAATGAAGCCTTACAGTTCCGTAATGAGGCTGAGACTTTCAGAAACCAAGCGGAGGGCTTTAAGAACGAGTCCAGTACCAACGCTACGAACACAAAGCAGTGGCGCGATGAGACCAAGGGTTTCCGAGACGAAGCCGAGCGGTTCAAGAATACGGCTGGTCAATACGCTACATCTGCTGGGAACTCTGCTTCCGCTGCGCATCAATc                                                                        <  1:462100/293‑1 (MQ=255)`  
                                                                                                                                                                                                                                        `aTTGTGAACCTAGAGAACGCAGTGGATTACCGCGATGCTGTTCCGTTTGGTCAACTAAAGACCATGGACCAGAACTCATGGCAAGCACGTAATGAATCCTTACAGTTCCGTAATGAGGCTGAGACTTTCAGAAACCACGCGGAGGGCTTTAAGAACGAGTCCAGTACCAACGCTACGAACACAAAGCAGTGGCGCGATGAGACCAATGGTTTCCGAGACGAAGCCGAGCGGTTCAAGAATACGGCTGGTCAATACGCTACATCTGCTGGGAACTCTGCTTCCGCTGCGCATCAATCTGAgg                                                                   <  1:307918/301‑1 (MQ=255)`  
                                                                                                                                                                                                                                         `acaggAACCTAGCGAACGCCGTGGATGACCGCGATGCTGTTCCGTTTGGTCAACTAAAGATCATGGACCAGAACTCATGGCAAGCACGTAATGAAGCCTTACAGTTCCGTAATGAGGCTGAGACTTTCAGAAACCAAGCGGAGGGCTTTAAGAACGAGTCCAGTACCAACGCTACGAACACAAAGCAGTGGCGCGATGAGACCAAGGGTTTCCGAGACGAAGCCAAGCGGTTCAAGAATACGGCTGGTCAATACGCTACATCTGCTGGGAACTCTGCTTCCGCTGCGCATCAATCTGAGGt                                                                  <  1:25460/297‑1 (MQ=255)`  
                                                                                                                                                                                                                                           `gtgAACCTAGCGAACGCCGTGGATGACCGCGATGCTGTTCCGTTTGGTCAACTAAAGACCATGGACCAGCACTCATGGCAAGCACGTAATGAAGCCTTACAGTTCCGTAATGAGGCTGTGACTTTCAGAAACCAAGCGGAGGGCTTTAAGAACGAGTCCAGTACAAACGCTACGAACACAAAGCCGTGGCGCGATGAGACCAAGGGTTTCCGAGACGAAGCCAAGCGGTTCAAGAATACGGCTGGTCAATACGCTACATCTGCTGGGAACTCTGCTTCCGCTGCGCATCAATCTGAGGtca                                                                >  1:104386/1‑299 (MQ=255)`  
                                                                                                                                                                                                                                             `gAACCTAGCGAACGCCGTGGATGACCGCGATGCTGTTCCGTTTGGTCAACTAAAGACCATGGACCAGAACTCATGGCAAGCACGTAATGAAGCCTTACAGTTCCGTCATGAGGCTGAGACTTTCAGAAACCAAGCGGAGGGCTTTAAGAACGAGTCCAGTACCAACGCTACGAACACAAAGCAGTGGCGCGATGAGACCAAGGGTTTCCGAGACGAAGCCAAGCGGTTCAAGAATATGGCTGGTCAATACGCTACATCTGCTGGGAACTCTGCTTCCGCTGCGCATCAATCTGAGGTAAAc                                                              <  1:443726/301‑1 (MQ=255)`  
                                                                                                                                                                                                                                               `aCCTAGCGAACGCCGTGGATGACCGCGATGCTGTTCCGTTTGGTCAACTAAAGACCATGGACCAGAACTCATGGCAAGCACGTAATGAAGCCTTACAGTTCCGTAATGAGGCTGAGACTTTCAGAAACCAAGCGGAGGGCTTTAAGATCGAGTCCAGTACCAACGCTACGAACACAAAGCAGTGGCGCGATGAGACCAAGGGTTTCCGAGACGAAGCCAAGCGGTTCAAGAATACGGCTGGTCAATACGCTACATCTGCTGGGAACTCTGCTTCCGCTGCGCATCAATCTGAGGTAAACGc                                                            <  1:362998/301‑1 (MQ=255)`  
                                                                                                                                                                                                                                                `ccTAGCTAACGCCGTGGATGACCGCGATGCTGTTCCGTTTGGTCAACTAAAGACCATGGACCAGAACTCATGGCAAGCACGTAATGAAGCCTTACAGTTCCGTAATGAGGCTGAGACTTTCAGAAACCAAGCGGAGGGCTTTAAGAACGAGTCCAGTACCAACGCTACGAACACAAAGCAGTGGCGCGATGAGACCAAGGGTTTCCGAGACGAAGCCAAGCGGTTCAAGAATACGGCTGGTCAATACGCTACATCTGCTGGGAACTCTGCTTCCGCTTCGCATCAATCTGAGGTAAACGcg                                                           >  1:2032/1‑300 (MQ=255)`  
                                                                                                                                                                                                                                                    `gCGAACGCCGTGGAGGACCGGGATGCTGTTCCGTTTGGTCAACTAAAGACCATGGACCAGAACTCATGGCAAGCACGTAATGAAGCCTTACAGTTCCGTAATGAGGCTGAGACTTTCAGAAACCAAGCGGAGGGCTTTAAGAACGAGTCCAGTACCAACGCTACGAACACAAAGCAGTGGCGCGATTAGACCAAGGGTTTCCGAGACGAAGCCAAGCGGTTCAAGAATACGGCTGGTCAATACGCTACATCTGCTGGGAACTCTGCTTCCGCTGCGCATCAATCTGAGGTAAACGCTgaga                                                       <  1:222319/301‑1 (MQ=255)`  
                                                                                                                                                                                                                                                    `aagagacaggTGGATGACCGCGATGCTGTTCCGTGTGGTCAACTAAAGACCATGGACCAGAACTCATGGCAAGCACGTAATGAAGCCTTACAGTTCCGTAATGAGGCTGAGACTTTCAGAAACCAAGCGGAGGGCTTTAAGAACGAGTCCAGTACCAACGCTACGAACACAAAGCAGTGGCGCGATGAGACCAAGGGTTTCCGAGACGAAGCCGAGCGGTTCAAGAATACGGCTGGTCAATACGCTACATCTGCTGGGAACTCTGCTTCCGCTGCGCATCAATCTGAGGTAAACGCTgaga                                                       <  1:174366/292‑1 (MQ=255)`  
                                                                                                                                                                                                                                                     `acagcGCCGTGGATGACCGCGATGCGGTTCCGTTTGGTCAACT‑AAGACAATGGACAAGAACTCATGGCAAGAACGTAATGAAGCCTGACAGTGCCGTAATGAGGCTGAGACTTTCAGAAACCAAGCGGAGGGCTTTAAGAACGAGTCCAGTACCAACGCTACGAACACAAAGCAGTGGCGCGATGAGACCAAGGGTTTCCGAGACGAAGCCGAGCGGTTCAAGAATACGGCTGGTCAATACGCTACATCTGCTGGGAACTCTGCTGCCGCTGCGCATCAATCTGAGGTAAACGCTGAGAAc                                                     <  1:332272/297‑1 (MQ=255)`  
                                                                                                                                                                                                                                                             `gTGGATGACCGCGATGCTGTTCCGTTTGGTCAACTAAAGACCATGGACCAGAACTCATGGCAAGCACGTAATGAAGCCTTACAGTTCCGTAATGAGGCTGAGACTTTCAGAAACCAAGCGGAGGGCTTTAAGATCGAGTCCAGTACCAACGCTACGAACCCAACGCAGTGGCGCGCTGAGACCAAGGGTTTCCGAGACGAAGCCAAGCGGTTCAAGAATCCGGCTGGTCAATACGATACATCTGCTGGGAACTCTGCTTCCGCTGCGCATCAACCTGAGGTAAACGCTGAGAACTCTGCca                                              >  1:40206/1‑301 (MQ=255)`  
                                                                                                                                                                                                                                                             `gTGGATGACCGCGATGCTGTTCCGTTTGGTCAACTAAAGACCATGGACCAGAACTCATGGCAAGCACGTAATGAAGCCTTACAGTTCCGTAATGAGGCTGAGACTTTCAGAAACCAAGCGGAGGGCTTTAAGAACGAGTCCAGTACCAACGCTACGAACACAAAGCAGTGGCGCGATGAGACCAATGGTTTCCGAGACGAAGCCAAGAGGTTCAAGAATATGGCTGGTCAATACGCTACATCTGCTGGGAACTCTGCTTCCGCTGCGCATCAATCTGAGGTAAACGCTGAGAACTCTGccc                                              >  1:3437/1‑300 (MQ=255)`  
                                                                                                                                                                                                                                                                `gATGACCGCAATGCTATTCCGTTTGGACAACTAAAGACCAAGGACCAGAACTTATGGCAAGCACGTAATGAAGCCTTACAGTTCCGTAATGAGGCTGAGACTTTCAGAAACCAAGCGGAGGGCTGTAAGAACGAGTCCAGAACCAACGCTACGAACACTAAGCAGTGGCTCGAGGAGACCAAGGGTTTCCGAGACGAAGCCGAGCGGTTCAAGAATACGGCTGGTCAATACGCTACATCTGCTGGGAACTCTGCTTCCGCCGCGCATCAATCTGAGGTAAACGCTGAGAACTCTGCCACAg                                           <  1:500295/301‑1 (MQ=255)`  
                                                                                                                                                                                                                                                                     `ccGCGATGCTGTTCCGTTTGGTCAACTAAAGACCATGGACCAGAACTCATGGCAAGCACGTAATGAAGCCTTACAGTTCCGTAATGATGCTGAGACTTTCAGAAACCAAGCGGAGGGCTTTAAGAACGAGTCCAGTACCAACGCTACGAACACAAAGCAGTGGCGCGATGAGACCAAGGGTTTCCGAGACGAAGCCGAGCGGTTCAAGACTACGGCTGGTCAATACGCTACATCTGCTGGGAACTCTGCTTCCGCTGCGCATCACTCTGAGGTAAACGCTGATAACTCTGCCACAGCATcc                                      >  1:151923/1‑301 (MQ=255)`  
                                                                                                                                                                                                                                                                             `gtGTTCCGTTTTGTCAACTAACGACCAGTGACCAGAACTCATGGCAAGCACGTAATGAAGCCTTACAGTTCCGTAATGAGGCTGAGACTTTCAGGAACCAAGCGGAGGGCTTTAAGAACGAGTCCAGTACCAACGCTACGAACACAAAGCAGTGGCGCGATGAGACCAAGGGTTTCCGAGACGAAGCCAAGCGGTTCAAGGATACGGCTGGTCAATACGCTACATCTGCTGGGAACTCTGCTTCCGCTGCGCATCAATCTGAGGTAAACGCTGAGAACTCTGCCACAGCATCCGCTAActa                              <  1:448578/300‑2 (MQ=255)`  
                                                                                                                                                                                                                                                                                         `gTCAACTAAAGACCATGGACCAGAACTCATGGCAAGCACGTAATGAAGCCTTACAGTTCCGTAATGAGGCTGAGACTTTCAGAAACCAAGCGGAGGGCTTTAAGAACGAGTCCAGTACCAACGCTACGAACACAAAGCAGTGGCGCGATGAGACCAAGGGTTTCCGAGACGAAGCCGAGCGGTTCAAGAATACGGCTGGTCAATACGCTACATCTGCTGGGAACTCTGCTTCCGATGCGCATCAATCTGAGGTAAACGCTGAGAACTCTGCCACAGCATCCACTAACTCTGCTCATCTgtc                  >  1:390229/1‑299 (MQ=255)`  
                                                                                                                                                                                                                                                                                           `aaaCTAAAGACCATGGACCAGAACTCATGGCAAGCACGTAATGAAGCCGTACAGTTCCGTAATGAGGCTGAGACTTTCAGAAACCAAGCGGAGGGCTTTAAGAACGAGTCCAGTACCAACGCTACGAACACAAAGCAGTTGCGCGATGAGACCAAGGGTTTCCGAGACGAAGCCAAGCGGTTCAAGAATACGGCTGGTCAATACGCTACATCTGCTGTGAACTCTGCTTCCGCTGCGCATCAATCTGAGGTAAACGCTGATAACTCTGCCACAGCATCCGCTAACTATGCTCATTTGGCAg                <  1:12732/300‑1 (MQ=255)`  
                                                                                                                                                                                                                                                                                             `aCTAAAGACCATGGACCAGAACTCATGGCAAGCACGTAATGAAGCCTTACAGTTCCGTAATGAGGCTGAGACTTTCAGAAACCAAGCGGCGGGCTTTAAGAACGAGTCCAGTACCAACGCTACGAACACAAAGCAGTGGCGCGATGAGACCAAGGGTTTCCGCGACGAAGCCAAGCGGTTCAAGAATATGGCTGGTCAATACGCTACATCTGCTGGGAACTCTGCTTCCGCTGCGCATCAATCTGAGTTAAACGCTGAGAACTCTGCCACAGCAGCCGCTAACTATGCTCATTTGGCAGaa              >  1:186345/1‑301 (MQ=255)`  
                                                                                                                                                                                                                                                                                             `aCTAAAGACCATGGACCAGAACTCATGGCAAGCACGTAATGAAGCCTTACAGTTCCGTAATGAGGCTGAGACTTTCAGAAACCAAGCGGAGGGCTTTAAGAACGAGTCCAGTACCAACGCTACGAACACAAAGCAGTGGCGCGATGAGACCAAGGGTTTCCGAGACGAAGCCGAGCGGTTCAAGAATACGGCTGGTCAATACGCTACATCTGCTGGGAACTCTGCTTCCGCTGCGCATCAATCTGAGGTAAACGCTGAGAACTCTGCCACAGCATCCGCTAACTATGCTCATTTGGCAGaa              >  1:428426/1‑301 (MQ=255)`  
                                                                                                                                                                                                                                                                                             `aCTAAAGACCATGGACCAGAACTCATGGCAAGCACGTAATGAAGCCTTACAGTTCCGTAATGAGGCTGAGACTTTCAGAAACCAAGCGGAGGGCTTTAAGAACGAGTCCAGTACCAACGCTACGAACACAAAGCAGTGGCGCGATGAGACCAAGGGTTTCCGAGACGAAGCCAAGCGGTTCAAGAATACGGCTGGTCAATACGCTACATCTGCTGGGAACTCTGCTTCCGCTGCGCATCAATCTGAGGTAAACGCTGAGAACTATGCCAAAGCATCCGCTAACTCTGCTCATTTGGCAGac              >  1:214003/1‑300 (MQ=255)`  
                                                                                                                                                                                                                                                                                             `aCTAAAGACCATGGACAAGAACTCATGGCAAGCACGTAATGAAGCCTTACAGTTCCGTAATGAGACTGAGACTTTCAGAAACCAAGCGGAGGGCTTTAAGTACGAGTCCAGTACCAACGCTACGAACACAAAGCAGTGGCGCGATGAGACCAAGGGTTTCCGAGACGAAGCCAAGCGGTTCAAGAATACGGCTGGTCAATACGCTACATCTGCTGGGAACTCTGCTTCCGCTGCGCATCAATCTGAGGTAAACGCTGAGAACTCTGCCACAGCATCCGCTAACTATGCTCATTTGGCAGaa              >  1:414186/1‑301 (MQ=255)`  
                                                                                                                                                                                                                                                                                              `cTAAAGACCATGGACCAGAACTCATGGCAAGCACGTAATGAAGCCTTACAGTTCCGTAATGAGGCTGAGACTTTCAGAAACCAAGCGGAGGGCTTTAAGAACGAGTCCAGTACCAACGCTACGAACACAAAGCAGTGGCGCGATGAGACCAAGGGTTTCCGAGACGAAGCCAAGCGGTTCAAGAATACGGCTGGTCAATACGCTACATCTGCTGGGAACTCTGCTTCCGCTGCGCATCAATCTGAGGTAAACGCTGAGAACTCTGCCACAGCATCCGCTAACTATGCTCATTTGGCAGAAc             <  1:51909/301‑1 (MQ=255)`  
                                                                                                                                                                                                                                                                                                    `aCCATGGACCAGAACTCCTGGCAAGCACGTAATGAAGCCTTACAGTTCCGTAATGAGGCCGAGATTTTCAGAAACCAACCGGAGGGCTTTAAGAACGAGGACAGCACAAACGCTACGAACACATAGCGGTGGCGCGATGAGACCAAGGGTTTCCGAGACGAAACCAAGCGGTTCAAGAATACGGCTGGTTAATACGCTACATCTGCTGGGAAATTTGCTTCCGCTGCGCATAAATATGAGATAAACGATGAGAACTCTTCCACATCATCCGCTAACTATGCTCATTTTGATGAAAagcacg       >  1:68159/1‑299 (MQ=255)`  
                                                                                                                                                                                                                                                                                                    `aCCATGGACCAGAACTCATGGCAAGCACGTAATGAAGCCTTACAGTTCCGTAATGAGGCTGAGACTTTCAGAAACCAAGCGGAGGGCTTTAAGAACGAGTCCAGTACCAACGCTACGAAAACAAAGCAGTGGCGCGATGAGACCAAGGGTTTCCGAGACGAAGCCAAGCGGTTCAAGAATACGGCTGGTCAATACGCTACATCTGCTGGGAACTCTGCTTCCGCTGCGCATCAATCTGAGGTAAACGCTGAGAACTCTGCCACAGCATCCGCTAACTATGCTCATTTGGCAGAACagcaag       >  1:322950/1‑301 (MQ=255)`  

                                                                                                                                                                                                                                                                                                          `|`                                                                                                                                                                                                                                                                                                             
`CTCTCTGACAAAGGCTTGGGGTCCAGCCGATGGCTACACGACCATCGAGTTACGTCGAGTAACCTCCACTACCGACCGATTGGTTGACTTTACGGATGGTTCAATCCTCCGCGCGTATGACCTTAACGTCGCTCAGATTCAAACGATGCACGTAGCGGAAGAGGCCCGTGACCTCACTACGGATACTATCGGTGTCAATAACGATGGTCACTTGGATGCTCGTGGTCGTCGAATTGTGAACCTAGCGAACGCCGTGGATGACCGCGATGCTGTTCCGTTTGGTCAACTAAAGACCATGAACCAGAACTCATGGCAAGCACGTAATGAAGCCTTACAGTTCCGTAATGAGGCTGAGACTTTCAGAAACCAAGCGGAGGGCTTTAAGAACGAGTCCAGTACCAACGCTACGAACACAAAGCAGTGGCGCGATGAGACCAAGGGTTTCCGAGACGAAGCCAAGCGGTTCAAGAATACGGCTGGTCAATACGCTACATCTGCTGGGAACTCTGCTTCCGCTGCGCATCAATCTGAGGTAAACGCTGAGAACTCTGCCACAGCATCCGCTAACTCTGCTCATTTGGCAGAACAGCAAGCAGAC  >  NC_001604.1/34797‑35394`

  

Alignment Legend

Aligned base mismatch/match (shaded by quality score): `ATCG/ATCG` `< 3 ≤` `ATCG/ATCG` `< 9 ≤` `ATCG/ATCG` `< 17 ≤` `ATCG/ATCG` `< 32 ≤` `ATCG/ATCG` `< 38 ≤` `ATCG/ATCG`

Unaligned base: `atcg`    Masked matching base: `atcg`    Alignment gap: `‑`    Deleted base: `‑`
